# Characteristics of Pupil Offset in Young Asian Adults With Mild-Moderate and High Myopia

**DOI:** 10.1167/tvst.11.6.13

**Published:** 2022-06-13

**Authors:** Shengshu Sun, Zhanglin Liu, Yuan Wu, Xiaowen Sun, Shaozhen Zhao, Yue Huang

**Affiliations:** 1 Tianjin Medical University Eye Hospital, College of Optometry, Institute of Ophthalmology, National Clinical Medical Research Center for Eye, Ear, Nose and Throat Diseases, Tianjin Branch, Tianjin Key Laboratory of Retinal Function and Diseases, Tianjin, China; 2 Department of Ophthalmology, People's Hospital of Rizhao, Rizhao, Shandong, China

**Keywords:** pupil offset, refractive surgery, oculus Pentacam, angle kappa

## Abstract

**Purpose:**

The purpose of this study was to explore the characteristics of pupil offset in young Asian adults with myopia.

**Methods:**

In total, 1200 eyes (600 young adults, 18–35 years old) were divided into mild-moderate and high groups according to equivalent spherical diopters (SEQ). The pupil offset and its X and Y components were compared between the groups. Linear correlation was analyzed among pupil offset, X and Y components, and SEQ. Multiple linear regression analysis was conducted for pupil offset and eye parameters.

**Results:**

The mean age of all subjects was 22.5 ± 4.8 years. The mean magnitude of the pupil offset (0.18 ± 0.09 mm vs. 0.15 ± 0.08 mm) and Y component (0.12 ± 0.08 mm vs. 0.10 ± 0.07 mm) were larger in the high group than in the mild-moderate group (*P* < 0.05). The magnitude of pupil offset, X and Y components, and SEQ were positively correlated. The pupil center (PC) of the right eye in the mild-moderate group was mainly superotemporal to the corneal vertex and mainly superonasal for the left eye and both eyes in the high group. Multiple linear regression analysis revealed that the magnitude of pupil offset correlated with central corneal thickness, intraocular pressure, and mean corneal curvature (*P* < 0.05).

**Conclusions:**

The magnitude of the pupil offset that correlated with partial eye parameters and its X and Y components increased as the SEQ increased, and the PC gradually shifted toward the superonasal direction in young Asian adults with myopia.

**Translational Relevance:**

Subjects with high myopia with a larger pupil offset should be considered for better postoperative visual quality during refractive surgery.

## Introduction

**Figure 1.**
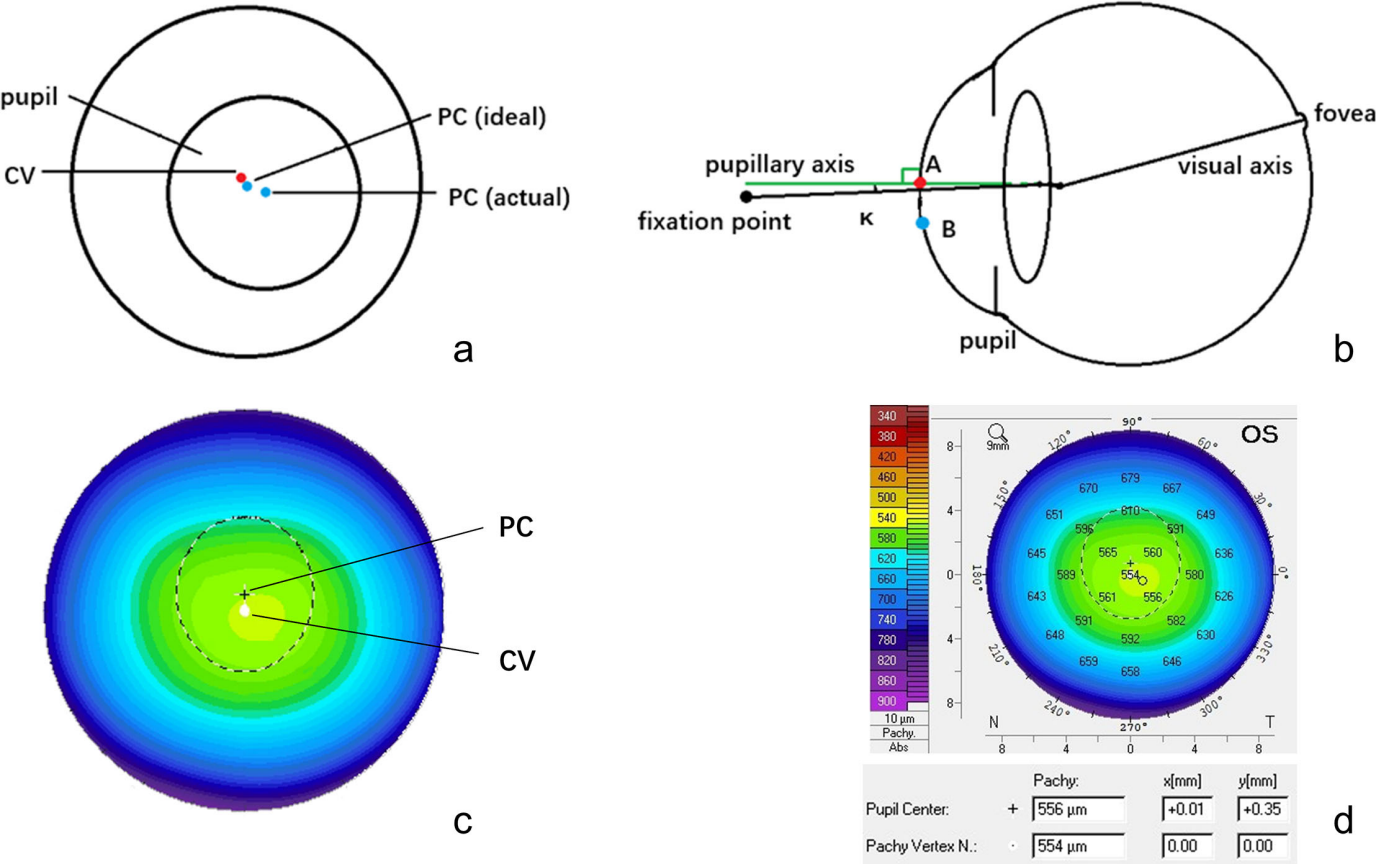
(**A**) Schematic sketch of pupil center (PC) and corneal vertex (CV), (**B**) schematic sketch of angle Kappa; red dot A: ideal position of PC, blue dot B: actual position of PC, (**C**), (**D**) X and Y components of pupil offset, PC, and CV measured by Pentacam.

The pupil offset is defined as the distance and direction between the entrance pupil center (PC) and corneal vertex (CV),^1^ which is a critically important eye parameter in clinical practice. CV is considered to be the best morphological approximation of the intersection of the visual axis and the corneal surface.^2^ PC and CV are theoretically coincident under ideal conditions; however, there is an offset between them due to the asymmetric optical system of the eye (Fig. 1A). Moreover, the angle kappa is a crucial parameter for characterizing the intersection angle of the visual axis and pupillary axis (Fig. 1B), which is not easily measured directly. The pupil offset and angle Kappa are similar concepts of the ocular anterior segment in clinical studies, which can be cross-referenced.^3^^,^^4^

Accurate centration is crucial in refractive surgery because decentered treatment may result in undesirable visual outcomes.^5^ Previous studies have shown that CV is an ideal ablation center with fewer postoperative higher-order aberrations (HOAs) introduced than PC.^6^^,^^7^ With the increase in HOAs, there are a series of complications related to postoperative visual qualities, such as poor night vision, glare, monocular diplopia, and astigmatism.^1^^,^^8^^,^^9^ Thus, it is essential to study the distribution of pupil offset in the myopic population to improve postoperative visual quality.

Relevant statistics^10^ have revealed that approximately 30% of the global population will develop myopia in 2020, possibly reaching 50% by 2050. The prevalence of myopia in young adults has progressively increased to 80% to 90%, which may be related to less outdoor time and increased educational pressures.^11^ Meanwhile, the progress of refractive surgery during the last 3 decades is appreciated by the majority of people.^12^ An increasing number of young adults who want to get rid of spectacles or contact lenses prefer refractive surgery. As a result, it is necessary to explore the pupil offset of young adults with myopia to design appropriate operational protocols that suit them.

This study aimed to explore the distribution of pupil offset in young Asian adults with different degrees of myopia and analyze the relationship between pupil offset and eye parameters. Although the pupil offset or angle kappa has been well studied in previous studies, different degrees of myopia have not been fully addressed, nor have they completely focused on young adults who are the primary group for refractive surgery. In contrast, this study concentrates on young adults with myopia, which can provide a supplementary study for the above clinical problems. It is clinically important to clarify the characteristics of the pupil offset in myopia. More importantly, it will provide a vital pre-operative reference for surgeons and raise attention to pupil offset in patients with different degrees of myopia. It further provides a suitable surgical design for refractive surgery in young adults and avoids postoperative decreases in visual quality caused by decentered ablation.

## Patients and Methods

### Patients

A total of 600 young patients with myopia (1200 eyes, 281 men and 319 women, aged 18–35 years) who underwent comprehensive pre-operative examination for refractive surgery were recruited for this cross-sectional study from January 2021 to August 2021 at Tianjin Medical University Eye Hospital.

According to the refractive surgery consensus, the inclusion criteria were as follows: (1) the diopter was stable for at least 2 years. (2) Soft contact lenses, hard contact lenses, and orthokeratology were discontinued at least 2 weeks, 1 month, and 3 months before the operation, respectively. (3) The best-corrected visual acuity (BCVA) was >20/25. The exclusion criteria were as follows: active inflammation and serious accessory organ lesions of the eyes, glaucoma, keratoconus, diabetes, serious mental illness, and autoimmune illness.

All recruited subjects appreciated the purpose and significance of this study, and written informed consent was obtained from them. The study was approved by the Ethics Committee of Tianjin Medical University Eye Hospital and conformed to the principles of the Declaration of Helsinki (2020KYL-33).

### Experimental Procedure

According to SEQ (range –0.75 D to –10.00 D), the subjects were divided into the mild-moderate myopia group (–0.75 D ≤ SEQ ≤ –6.00 D) and the high myopia group (–6.25 D ≤ SEQ ≤ –10.00 D). Comprehensive pre-operative ophthalmic examinations were performed for all subjects, including uncorrected visual acuity (UCVA), BCVA, dominant eye, intraocular pressure (non-contact tonometer, TX-F, Canon), slit-lamp examination, dilated fundus examination, apparent optometry, corneal topography, pupil offset, pupil diameter, corneal curvature, and corneal thickness. All examinations and data collection were performed by skilled ophthalmologists and operators under the same conditions.

### Measurement of Pupil Offset and its X and Y Components

As stated by Gharaee,^13^ X-component and Y-component were used to represent the horizontal and vertical components of pupil offset for further study of pupil offset, respectively. Pupil offset and its X and Y components could be directly and automatically measured by the Pentacam HR (Oculus Optikgeräte GmbH, Wetzlar, Germany), which can accurately and repeatedly scan the anterior segment of the eyes presented in previous studies (Figs. 1C, 1D).^14^^,^^15^ The Pentacam scanned the anterior and posterior corneal surfaces by 360 degrees rotation within 2 second through a rotating Scheimpflug camera with a short wavelength slit-light, and 25 corneal Scheimpflug images, and a three-dimensional reconstruction of the cornea was obtained.

Before the measurements, the head positions of the participants were suitably adjusted. During the measurement, the subjects were strongly urged to keep their eyes open, look at the target of the device, and not blink. After the measurement, all subjects were required to close their eyes for a few moments to ensure an even distribution of the tear film for the next examination. Data were accepted only when the quality specification showed OK; otherwise, it was remeasured. The pupil offset was ideally measured three times per eye with eligible data, and the mean value of the three measurements was taken as the final pupil offset of each patient to ensure the reliability of the data.

### Statistical Analysis

IBM SPSS Statistics 20 (IBM, Armonk, NY, USA) and Prism 9.0 (GraphPad Software) were used for data processing and statistical analysis. The Kolmogorov–Smirnov test was used to test the normality of the data, which are presented as mean ± standard deviation (SD). The UCAV, SEQ, pupil offset, and its X and Y components were compared between the two groups using the generalized estimation equation (GEE), which could be used to analyze nonindependent data measured from both eyes of each patient. Scatter plots (CV as the zero point) with a confidence ellipse were used to describe the distribution of pupil offset in 1200 eyes and to determine the orientation with the greatest SD. A linear correlation analysis was conducted for the pupil offset, X and Y components, and SEQ. In addition, multiple linear regression analyses (stepwise) based on reasonable independent variable screening mechanisms were performed to identify the parameters that were correlated with the magnitude of pupil offset and its X and Y components. Values of *P* < 0.05 were considered statistically significant.

## Results

### Demographic Data and Pupil Offset of the Study Population

The baseline characteristics and results of 600 subjects with 1200 eyes are shown in Table 1 to avoid confounding bias. The mean ages for the mild-moderate myopia group and high myopia group were 22.2 ± 4.8 and 22.9 ± 4.9 years old, respectively (*P* > 0.05, independent-samples *t*-test; see Table 1). There were no differences in sex or the dominant eye (*P* > 0.05, chi-square test; see Table 1). UCVA, SEQ, the magnitude of pupil offset, and its X and Y components were compared between the mild and moderate and high myopia groups using GEE (Table 2). The UCAV and SEQ of the two groups showed clear statistical differences (*P* < 0.01). The mean pupil offset of the high myopia group (0.18 ± 0.09 mm) was larger than the mild-moderate myopia group (0.15 ± 0.08 mm, *P* < 0.05). Further comparison of the X and Y components was performed via GEE, and the mean value of the Y-component was larger in the high group (0.12 ± 0.08 mm) than in the mild-moderate group (0.10 ± 0.07 mm) with a significant difference (*P* < 0.05), whereas there was no significant difference in the X-component (*P* > 0.05).

**Table 1.** Comparisons of Baseline Characteristics Between the Mild-Moderate and High Myopia Groups (Mean ± SD).

Group	Mild-Moderate	High	*X^2^*/t Value	*P* Value
Cases/eyes	300/600	300/600		
Gender (male/female, n/n)	145/155	136/164	0.542	0.462
Dominant eye (right/left, n/n)	192/108	186/114	0.257	0.612
Age/years	22.2 ± 4.8	22.9 ± 4.9	−1.803	0.072

**Table 2.** Comparisons of UCVA, SEQ, Magnitude of Pupil Offset, and its X and Y Components Between the Mild-Moderate Myopia Group and the High Myopia Group (Mean ± SD)

Variations	M ± SD	β Value	SE	95% Wald CI	Wald Chi-Square	*P* Value
(intercept)	(LogMAR)	1.354	0.0209	1.313−1.395	4190.37	0
UCVA of mild-moderate group	0.86 ± 0.36	−0.395	0.0253	−0.444−0.345	244.357	0.001^*^
UCVA of high group	1.31 ± 0.28	0^a^				
(intercept)	(D)	−7.734	0.0744	−7.88 −7.588	10816.453	0
SEQ of mild-moderate group	−3.28 ± 1.29	3.837	0.0877	3.665 − 4.008	1915.68	0.001^*^
SEQ of high group	−7.44 ± 0.96	0^a^				
(intercept)	(mm)	0.166	0.0059	0.155 − 0.178	782.582	0
Pupil offset of mild-moderate group	0.15 ± 0.08	−0.011	0.005	−0.021 −0.002	5.197	0.023^*^
Pupil offset of high group	0.18 ± 0.09	0^a^				
(intercept)	(mm)	0.094	0.0055	0.084 − 0.105	291.168	0
X component of mild-moderate group	0.10 ± 0.07	−0.005	0.0044	−0.014 − 0.004	1.366	0.243
X component of high group	0.11 ± 0.08	0^a^				
(intercept)	(mm)	0.123	0.0053	0.113 − 0.134	544.669	0
Y component of mild-moderate group	0.10 ± 0.07	−0.011	0.0046	−0.02 −0.002	5.722	0.017^*^
Y component of high group	0.12 ± 0.08	0^a^				

a Set to 0 due to redundancy of this parameter.

* Significant difference, *P* < 0.05 (The following are the same)

Abbreviations: SE, standard error; CI, confidence interval; LogMAR, logarithm of the minimum angle of resolution; D, diopter.

### Histogram of the Pupil Offset Magnitude

**Figure 2.**
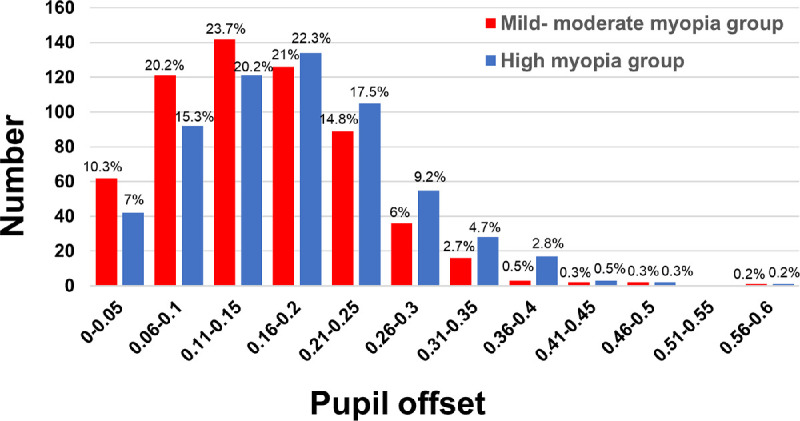
Pupil offset magnitude of mild-moderate and high myopia group.

Figure 2 presents the distribution of pupil offset magnitude in the mild-moderate and high myopia groups. Among these, 451 eyes (75.2%) in the mild-moderate myopia group and 389 eyes (64.8%) in the high myopia group had pupil offset smaller than 0.2 mm; 595 eyes (99.2%) in the mild-moderate myopia group and 594 eyes (99.0%) in the high myopia group had a pupil offset that was smaller than 0.4 mm. Overall, the pupil offset magnitude in subjects with high myopia was greater than that in subjects with mild-moderate myopia.

### Linear Correlation Analysis of Pupil Offset and SEQ

**Figure 3.**
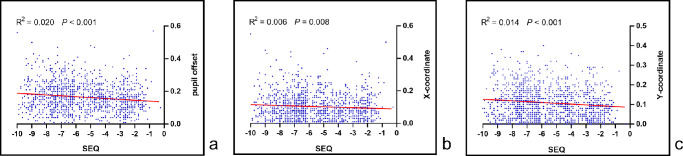
Linear correlation analysis between the magnitude of (**A**) pupil offset, (**B**) X-component, (**C**) Y-component and SEQ.

As shown in Figure 3A, there was a positive correlation between the pupil offset and SEQ. The higher the pre-operative SEQ, the larger the pupil offset magnitude (*P* < 0.001). Similarly, as the SEQ increased, the Y-component (Fig. 3C) of the pupil offset magnitude increased (*P* < 0.001) compared with the X-component (*P* = 0.008; Fig. 3B). In addition, compared with the X-component (R^2^ = 0.006), the Y-component had a stronger correlation with the SEQ (R^2^ = 0.014).

### Scatter Plots of the Pupil Offset

**Figure 4.**
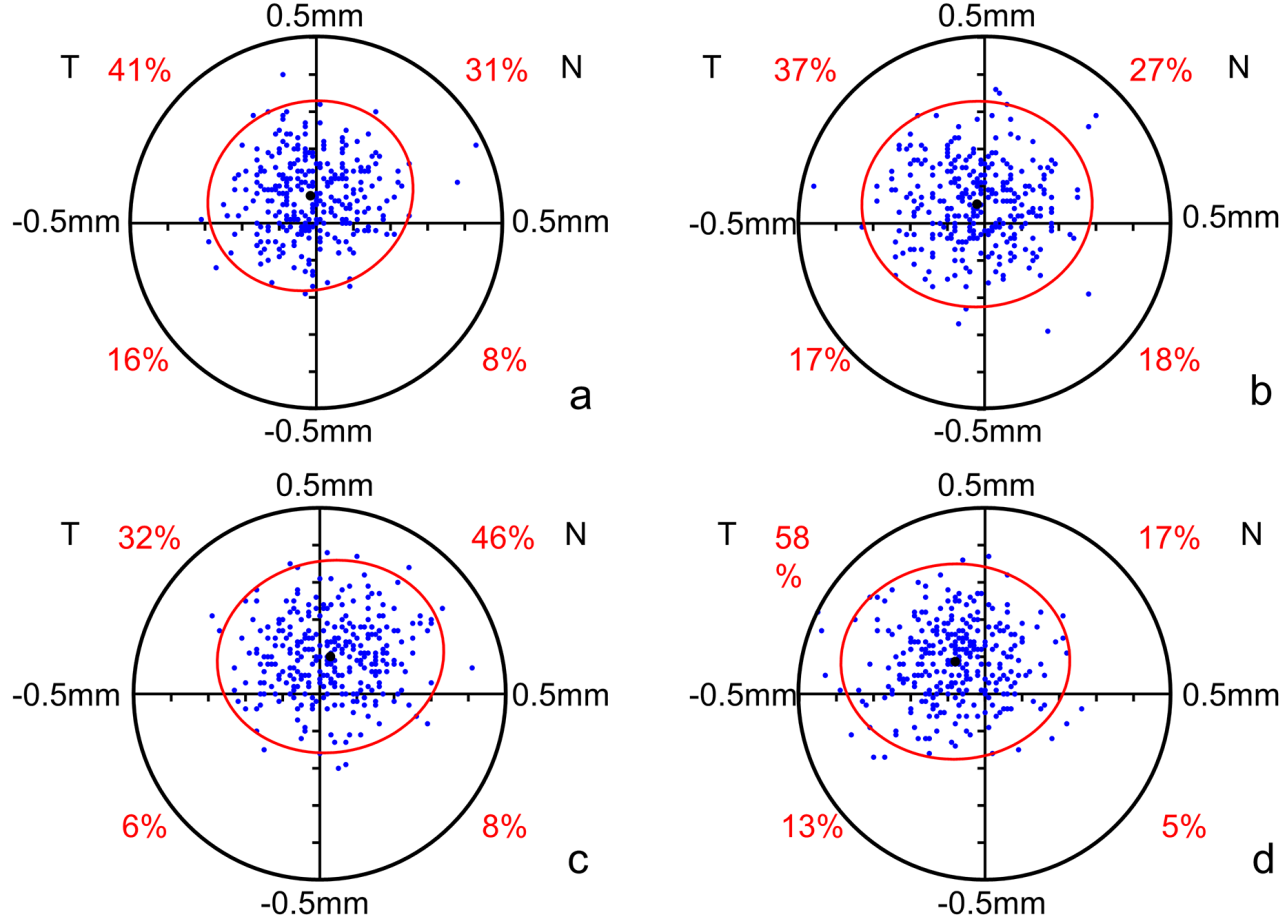
Scatter plots of pupil offset direction of (**A**) the right eyes and (**B**) the left eyes in the mild-moderate myopia group. Scatter plots of pupil offset direction of (**C**) the right eyes and (**D**) the left eyes in the high myopia group. The blue dots represent the PC of each eye. The red ovals represent the standard deviation ellipses. The black dots represent the vector means. Abbreviations: T, temporal; N, nasal.

As shown in Figure 4, a coordinate system in which the blue dots represent the PC was established, centering on the CV traced by Pentacam. The four quadrants represent the superior, inferior-temporal, and nasal areas. The scatter plot (Fig. 4A) indicated that the PC of 72% of the right eyes in the mild-moderate myopia group was superior to that of the CV, especially in the superotemporal position (41%). The PC of 64% of the left eyes was also superior to the CV, especially in the superonasal area (37%; Fig. 4B). In the high myopia group, the PC of 78% of the right eyes and 75% of the left eyes were superior to the CV, and both were predominantly superonasal (46% and 58%, respectively; Figs. 4C, 4D). The eyes of the high myopia group showed a distribution that was more concentrated in the superonasal quadrant than those in the mild-moderate myopia group.

### Multiple Linear Regression Analysis (Stepwise) of Pupil Offset and its X and Y Components

The results of the multiple linear regression analysis (stepwise) are summarized in Table 3 to avoid the effect of statistically insignificant variables on the regression analysis for accurate results. Correlations between the magnitude of pupil offset and scotopic pupil diameter, central corneal thickness (CCT), intraocular pressure (IOP), and mean corneal curvature were analyzed using multiple regression analysis. The results showed that pupil offset was negatively correlated with IOP and mean corneal curvature (*P* < 0.05; Table 3), and positively correlated with CCT (*P* < 0.05). Multicollinearity was weak between the parameters, based on the values of variance inflation factor (VIF) around 1.000.

**Table 3.** Multiple Linear Regression Analysis (Stepwise) of Pupil Offset and its X and Y Components With Partial Ocular Parameters and Age

	Pupil Offset		X-component		Y-component		
Parameters	Coefficient	*P* Value	Coefficient	*P* Value	Coefficient	*P* Value	VIF
Scotopic pupil diameter	−0.01	0.135	−0.003	0.563	0.001	0.844	1.000
CCT	0.001	0.01*	0.001	0.501	0.001	0.432	1.000
IOP	−0.004	0.001*	0.001	0.676	0.001	0.699	1.002
Mean corneal curvature	−0.004	0.038*	0.001	0.600	−0.001	0.629	1.007
Age	0.001	0.560	0.001	0.682	−0.001	0.175	1.000

Abbreviations: VIF, variance inflation factor; CCT, central corneal thickness; IOP, intraocular pressure.

## Discussion

The human eye is a complex optical system with various essential optical parameters, such as pupil offset, visual axis, pupillary axis, and angle kappa. Pupil offset, considered to be the decentration between PC and CV, can lead to decentered ablation in refractive surgery.^1^ With the detailed development of research, the choice of ablation center gradually changed from the preceding PC to the now widely accepted CV.^7^ The aberration introduced by the pupil offset is mainly HOA,^16^ which may be due to the pupil offset in the center of the Zernike tree. Fewer HOAs and better postoperative visual quality are introduced when the ablation center is located at the CV rather than at the PC.^9^^,^^17^ Therefore, the characteristics of pupil offset in young Asian adults with different degrees of myopia, who are the primary population of refractive surgeries, were investigated in this study for reference.

Pentacam is used to measure the pupil offset and X and Y components in this study as Frings’ procedure, and accurate results of pupil offset can be obtained with this method.^18^^,^^19^ Although there are various instruments for measuring pupil offset in clinical practice, a large number of clinical applications and studies have shown that these instruments can accurately measure pupil offset.^20^ However, we should be careful when comparing results from various instruments due to the different principles of each instrument.

The magnitude of pupil offset in the high myopia group was significantly larger than that in the mild-moderate myopia group, which was different from previous studies. For instance, Reinstein et al. found that the largest pupil offset was in the hyperopia group and the smallest was in the myopia group.^4^ Hashemi et al. suggested that the largest pupil offset was in the emmetropia group, and the smallest was in the myopia group.^21^ The proposed reason why the smallest pupil offset was in the myopia group can be attributed to geometric features of the eyes. The statistical results of this study suggest that the influence of other factors on pupil offset in subjects with myopia should be considered. First, the optometric characteristics of patients with different degrees of myopia should be considered. Lee et al. found that subjects with higher myopia or longer axial length had a closer distance with the work content during near work.^22^ Compared to the mild-moderate myopia group, high myopia's shorter work distance can increase the angle of the visual axis between the right and the left eyes, thus leading to the visual axis shifting to the nasal side. Meanwhile, the pupil assembly reflex persisted for a longer time in patients with high myopia, which may have caused the PC to be nasal and a larger pupil offset. Second, high myopia was prone to pathological myopia with corresponding ocular fundus changes, such as posterior scleral staphyloma, macular choroidal atrophy, myopic choroidal neovascularization, and macular retinal splitting.^23^ These ocular fundus lesions may not significantly affect visual acuity but may cause a macular shift, leading to the displacement of the visual axis and separation of the visual axis and pupillary axis, thus producing large angle kappa and pupil offset. Last, the region of the studied population was considered a factor. The participants included in this study were young adults from northern China. Therefore, the results of this study may be inconsistent with those of previous studies that concentrated on Western populations or a population with a larger age range. There are regional and refractive-error-specific differences in pupil offset; thus, no generalizations can be made.

The pupil offset was linearly and positively correlated with the preoperative SEQ, which was inconsistent with Reinstein et al., who stated that pupil offset in subjects with myopia decreased as the SEQ increased.^4^ This inconsistency can be explained by the age of the included study population. It has been shown that pupil offset decreases with increasing age.^13^ This study focused on young adults with a mean age of 22.5 ± 4.8 years, whereas the population studied by Reinstein et al. had a relatively large age span, ranging from 18 to 69 years with a mean age of 40 ± 10 years.

The Y-component of pupil offset in the high myopia group was greater than that in the mild-moderate myopia group. The difference in the Y-component between the two groups was similar to the findings of Holden et al.,^23^ which can be attributed to the longer axial length and macular shift caused by high myopia. In addition, this study proposed that the greater Y-component in high myopia than in mild-moderate myopia might be caused by the aforementioned optometric features and ocular fundus-related diseases in patients with high myopia. Moreover, the Y-component was more strongly correlated with the SEQ than was the X-component, indicating that the Y-component exerted a greater effect on the magnitude of the pupil offset than did the X-component. Consistent with our results, Li et al. and Padmanabhan et al. reported that decentration was predominantly along the vertical axis.^3^^,^^24^ However, Reinstein et al.^4^ found that the Y-component was not linearly correlated with the SEQ, which might be due to differences in race, age, or sample size. More clinical studies need to be performed on the detailed characteristics of the X and Y components of the pupil offset and its effects on HOAs.

In the mild-moderate myopia group, the PC was predominantly temporal to the CV in the right eyes and nasal to the CV in the left eyes, which can be explained by the aforementioned right-handedness. In the high myopia group, the PC of both the right and left eyes was predominantly nasal to the CV, which was similar to a study that found that the PC gradually shifted to the nasal side of the CV as the axial length increased.^25^ This may be attributed to a near shift of vision and refractive internal strabismus in high myopia.^26^ Closer vision caused a convergence reflex in both eyes, resulting in a shift of the PC to the nasal side. This study also found that most PCs were above CVs. A different result was reported by Hashemi et al.,^21^ who found that in 400 eyes, 90.2% of PCs were inferior to CVs. A factor contributing to this difference was that this study included both eyes, whereas Hashemi et al. only included the right eyes.

In this study, 40.67% (488) of eyes had a PC that deviated toward the temporal side of the CV, 56.92% (683) of eyes had a PC that deviated toward the nasal side, and 43% (516) of eyes had a superonasal tendency, consistent with Chung et al.,^1^ who found that the superonasal tendency of decentration was predominant among Korean subjects, attributing to the parallax error measured by Pentacam. Different results were reported by Reinstein et al.^4^ who found that 85.2% of 250 myopic eyes had PC deviation to the temporal side, and by Mabed et al.,^27^ who found that over 80% of 248 myopic eyes had PC deviation to the temporal side. First, more subjects with high myopia who had a PC nasal to the CV, as described above, were included in this study than in other studies, which may be one reason for this difference. Moreover, there are differences in Asian and European human eye parameters owing to ethnic differences. The small sample size may also account for this difference.

Multiple linear regression analysis suggested that the pupil offset correlated with CCT, IOP, and mean corneal curvature. The negative correlation between the pupil offset and IOP may be attributed to the fact that increased IOP can lead to changes in pupil and corneal surface morphology, which in turn decreases pupil offset. There was no correlation between pupil offset and age, which differed from the study of Hashemi et al., which may be due to the age range of the patients in this study.^21^ The effect of the mean corneal curvature may be related to geometric features of the eyes. Studies have shown that the magnitude of pupil offset increases as the pupil becomes larger.^4^^,^^27^ The pupil in this study was measured under darkroom conditions with no significant change, and no relationship was found between different dark pupil diameters and pupil offset magnitude.^28^ In addition, the correlation coefficients among CCT, IOP, mean corneal curvature, and pupil offset were small, and the variation in those among different myopia degrees was relatively small; thus, further studies are needed.

A limitation of this study is that pupil offset, CV, and PC were tested using devices based on a two-dimensional screen; however, refractive surgery is a three-dimensional procedure. Further studies on the three-dimensional structure of the pupil offset should be conducted. In addition, only partial ocular anterior segment parameters that might be associated with pupil offset were included in the multiple linear regression analysis, and additional parameters should be investigated in further studies.

In conclusion, this study found that pupil offset in myopic Asian eyes of young adults increased with increasing pre-operative SEQ, indicating that pupil offset was greater in subjects with high myopia than in those with mild-moderate myopia. In addition, as the SEQ increased, the PC gradually shifted toward the nasal side of the CV. To improve postoperative visual quality, refractive surgery must be well designed and corrected before and during the surgery due to the presence of pupil offset, especially in young adults with high myopia.
